# Supplementary material for: A Bifunctional Electrocatalyst for Oxygen Evolution and Oxygen Reduction Reactions in Water
Source: Angew Chem Int Ed Engl. 2016 Jan 15;55(7):2350–5. doi: 10.1002/anie.201508404 (PMC4949709; doi:10.1002/anie.201508404)
Supplement: Supplementary file 1 — Supplementary [file ANIE-55-2350-s001.pdf]

Angewandte Chemie
Eine Zeitschrift der Gesellschaft Deutscher Chemiker

Supporting Information

A Bifunctional Electrocatalyst for Oxygen Evolution and Oxygen Reduction Reactions in Water

Wolfgang Schöfberger, Felix Faschinger, Samir Chattopadhyay, Snehadri Bhakta, Biswajit Mondal, Johannes A. A. W. Elemans, Stefan Müllegger, Stefano Tebi, Reinhold Koch, Florian Klappenberger, Mateusz Paszkiewicz, Johannes V. Barth, Eva Rauls, Hazem Aldahhak, Wolf Gero Schmidt, and Abhishek Dey**

anie_201508404_sm_miscellaneous_information.pdf

Experimental Section

Materials

All chemicals were commercially available and of the highest purity grade and used without further purification. Tetrabutyl ammonium perchlorate (TBAP), potassium hexafluorophosphate (KPF_6) and the solvents used in electrochemical experiments were purchased from Sigma Aldrich. Disodium hydrogen phosphate dihydrate ($Na_2HPO_4.2H_2O$), potassium chloride (KCl), potassium nitrate (KNO_3) and sodium hydroxide (NaOH) were purchased from Merck. Other used chemicals were purchased either from Sigma Aldrich or Acros chemicals or Alfa Aesar. Reagent grade solvents were purchased from Fischer chemicals and distilled prior to use. THF was distilled over sodium and benzophenone under an argon atmosphere and stored over molecular sieves (4 Å) upon use. 1-Phenyloctane used for the liquid-STM measurements was vacuum distilled prior to use. TLC was performed by using Fluka silica gel (0.2 mm) on aluminium plates. Silica-gel columns for chromatography were prepared with silica gel 60 (0.060–0.20 mesh ASTM) from Acros. Edge plane graphite (EPG) electrodes were purchased from Pine Instruments, USA. Highly oriented pyrolytic graphite was purchased from NT-MDT (ZYB grade, 1 cm^2).

Instrumentation

Proton (^1H NMR) and carbon (^{13}C NMR) spectra were recorded on a Bruker Ascend 700 spectrometer equipped with a cryogenically cooled probe (TXI), or on a Bruker Avance 500 MHz spectrometer. ^{19}F NMR spectra were recorded on a Bruker Avance 300 MHz at 282.4 MHz. The chemical shifts are given in parts per million (ppm) on the delta scale (δ) and are referenced to the residual non-deuterated solvent for ^1H and TFA for ^{19}F. MALDI-TOF measurements were collected with a Bruker Autoflex III Smartbeam spectrometer and on an Agilent atmospheric pressure photoionization (APPI) source on an Agilent 6520 quadrupole time-of-flight (QTOF) in the positive mode. UV-Vis absorption spectra were measured on a Varian CARY 100 Bio spectrophotometer. All electrochemical experiments were performed using a CH instruments (CHI720D) electrochemical analyzer. Bipotentiostat, reference electrodes, and counter electrodes were purchased from CH instruments. RRDE data was collected using the RRDE setup from Pine Research Instrumentation (E6 series with Changedisk tips with AFE6M rotor)

Construction of modified electrodes

Physisorption of the catalyst on EPG electrode: 100 µL of 1 mM catalyst in chloroform was deposited on a freshly cleaned EPG electrode mounted on a RRDE setup. Once solvent was fully evaporated, the surface was thoroughly dried with N_2 gas and rinsed with chloroform

and ethanol. Finally, the surface was rinsed with triply distilled water before using it in electrochemical experiments.

Cyclic Voltammetry experiments

All homogeneous CV experiments were performed in acetonitrile solution containing 1 mM catalyst and 100 mM TBAP (supporting electrolyte). All heterogeneous CV experiments were performed in buffer solution as mentioned. Buffer solution contained 100 mM Na_2HPO_4 and 100 mM KPF_6. The mentioned pH was adjusted by adding NaOH and H_3PO_4. In all cases platinum and Ag/AgCl were used as counter electrodes and reference electrodes, respectively.

Coverage Calculation

The coverage for a particular species was determined by integrating the oxidation and reduction currents of the respective species.[1,2]

Partially Reduced Oxygen species

A Platinum ring was polished first by alumina powder of grit size of 1, 0.3, 0.05 µm and then electrochemically cleaned. An EPG electrode was also cleaned by polishing and both were inserted into the RRDE tip. Then the RRDE tip was mounted on the rotor and immersed into a cylindrical glass container equipped with platinum and Ag/AgCl electrodes. The collection efficiency (CE) of the platinum in the RRDE setup was estimated in a 2 mM $K_3Fe(CN)_6$ and 100 mM KNO_3 solution at a rotation speed of 300 rpm and 10 mVps scan rate. Generally, a 15 ± 2% CE was observed during these experiments. For the detection of H_2O_2 by these experiments platinum was held at a constant potential was taken from literature.[1–3] For normal measurement of PROS, the ratios of ring and disk currents were taken at the potential where the Pt ring electrode exhibits the maximum current during RRDE experiments.

STM of manganese corroles on Ag(111) and HOPG

For the single-molecule investigations presented here, we chose a single-crystal surface of the silver as substrat. The Ag(111) substrate was prepared by cycles of Ar^+ ion sputtering (600 eV) and thermal annealing at 720 K. Mn(TpFPC) was sublimated at ultra-high vacuum conditions (base pressure $<10^{-9}$ mbar) from a quartz crucible at 500 K onto the substrate kept at room temperature and subsequently annealed at 360 K for 10 minutes. After pre-cooling to 100 K, the samples were in-situ transferred into the STM chamber. STM experiments were performed at 5 K and a base pressure of $<10^{-10}$ mbar employing electrochemically etched tungsten tips that were thermally deoxidized by flash-annealing to

above 1070 K. STM images were analyzed using the program WSxM.[4] The *dI/dV* signal was obtained with lockin technique adding a sinusoidal modulation (V=20 mV and v=757 Hz) to the dc bias. Total-energy density functional calculations (DFT) have been performed using the Vienna Ab Initio Simulation Package[5] including the projector-augmented wave method[6] for describing electron-ion interactions and the generalized gradient approximation (PW91 functional)[7] for modeling electron-electron exchange-correlation interactions. STM simulations were obtained via the Tersoff-Hamann model.[8] The STM measurements at the solid-liquid interface were performed at the HOPG-1-phenyloctane interface using a home-built liquid-STM.[9] A 10 µL droplet of manganese corrole solution ([**2**] =10^{-5} M) was brought onto a freshly cleaved HOPG surface and the STM tip (Pt/Ir 80/20) was immersed in it.

Synthesis Procedures

1) The synthesis of 5,10,15-tris(pentafluorophenyl)corrol was performed according to [1].

2) *Synthesis of 5,10-bis(pentafluorophenyl)-15-(2,3,5,6-tetrafluoro-4-((carboxyethyl)thio)-phenyl) corrole, 10,15-bis(pentafluorophenyl)-5-(2,3,5,6-tetrafluoro-4-((carboxyethyl)-thio)-phenyl) corrole and 5,15-bis(pentafluorophenyl)-10-(2,3,5,6-tetrafluoro-4-((carboxyethyl)-thio)-phenyl) corrole.*[2]

11.5 mg H_3TpFPC (0.014 mmol) and 10 equiv. of 60% sodium hydride in mineral oil were placed in a 3-necked round-bottom flask under argon. THF and 1 equiv. 3-mercaptopropionic acid were added and the reaction mixture was stirred at room temperature for 25 min. The reaction was monitored by TLC and water was added to the mixture after complete conversion. Saturated NH_4Cl was added to the water/THF-phase and the solution was extracted twice with DCM. The combined organic phases were extracted three times with water. The solvent was evaporated under reduced pressure. Purification by silica gel chromatography and preparative HPLC was performed (DCM:MeOH = 10:1) and the first fluorescent band was collected. Removal of the solvent under reduced pressure afforded 4.3 mg (40 % yield) mono-functionalized corrole. During the reaction 3,3 mg H_3TpFPC (29 %) and 3,0 mg (24 %) bi-functionalized corrole were obtained. Only traces of tri-functionalized corrole could be detected.

The regioselectivity of the S_NAr reaction can be quantified by determining the isomeric ratio $I_R = I_{5/15}/I_{10}$. The procedure of determining the isomeric ratio is exemplified for the mono-substituted corrole and is illustrated in Figure S1. The isomeric ratio is calculated by integrating the *o**-F resonances of the functionalized *meso*-tetrafluorenated phenyl

moiety and a value of $I_R = I_{5/15}/I10 = 3.9$ was calculated for the above stated purified reaction product.

Analytical data: ^1H NMR (300 MHz, $CDCl_3$, 30 °C): δ = 9.03 (s, 0.4H, H_β), 8.91 (s, 1.6H, H_β), 8.79 (d, J = 4.5 Hz, 1H, H_β), 8.73 (d, J = 4.2 Hz, 1H, H_β), 8.62 (d, J = 4.5 Hz, 0.4H, H_β), 8.55 (d, J = 4.4 Hz, 1.6H, H_β), 8.50 (m, 2H, H_β), 3.51 (m, 2H, CH_2), 2.98 ppm (m, 2H, CH_2); ^{13}C NMR (176.1 MHz, $CDCl_3$, 30 °C): δ = 174.6, 148.1, 147.4, 146.9, 146.7, 146.0, 145.6, 145.2, 142.7, 142.5, 142.0, 141.3, 140.6, 138.7, 138.5, 137.3, 137.0, 134.7, 134.3, 130.5, 130.3, 127.8, 126.4, 126.2, 121.8, 121.6, 117.4, 117.3, 99.2, 98.5, 95.5, 94.6, 42.8, 34.9 ppm; ^{19}F NMR (282.4 MHz, $CDCl_3$, 30 °C): δ = -133.49 (dd, ^3J = 25.0 Hz, ^4J = 11.8 Hz, 1.6F, F_o^*), -133.88 (dd, ^3J = 25.3 Hz, ^4J = 12.2 Hz, 0.4F, F_o^*), -137.05 - -137.28 (m, 2F, F_m^*), -137.71 - -137.80 (m, 4F, F_o), -152.25 (t, J = 20.9 Hz, 1.2F, F_p), -152.82 (t, J = 20.9 Hz, 0.8F, F_p), -161.48 (dt, ^3J = 22.2 Hz, ^4J = 7.5 Hz, 2.4F, F_m), -161.94 ppm (dt, ^3J = 22.5 Hz, ^4J = 7.6 Hz 1.6F, F_m); MS (MALDI+) m/z: calcd. for $C_{40}H_{16}F_{14}N_4O_2S$ 882.0770 $[M]^+$, 883.0849 $[M+H]^+$, found: 882.0825 $[M]^+$, 883.0846 $[M+H]^+$. HR-MS (ESI^-) : m/z: calcd. for $C_{37}H_{10}F_{15}N_4$: 795.0744; found: 795.0741 $[M-H]^-$; UV-Vis (THF) λ_{max}= 408, 418(sh), 563, 604 nm.

General procedure for metalation reactions:
Free base corroles (0.014 mmol) are dissolved in 3 mL DMF, 34.3 mg (140 µmol, 10 equiv.) manganese(II) acetate tetrahydrate were added and the reaction mixture was refluxed for 30 min under aerobic condition. The DMF was evaporated under reduced pressure and purified *via* column chromatography (silica gel, DCM:MeOH = 2:1). The products **1-3** were obtained in 79, 84, and 80 % yield, respectively.

Manganese 5,10-bis(pentafluorophenyl)-15-(2,3,5,6-tetrafluoro-4-((carboxyethyl)thio)-phenyl) corrole and *manganese 5,15-bis(pentafluorophenyl)-10-(2,3,5,6-tetrafluoro-4-((carboxy-ethyl)thio)phenyl)corrole* **1**.
Analytical data: yield = 79%, ^1H NMR (700 MHz, $CDCl_3$, 30°C): 20.13 (vbs, 1H), 8.95 (bs, 4H), 8.11 (bs, 4H), -0.79 (vbs, 1H), -17.73 (vbs, 1H), -41.12 ppm (vbs, 1H); ^{13}C NMR (175 MHz, $CDCl_3$): δ = 212.98, 151.84, 150.50, 146.83, 145.40, 142.77, 141.45, 140.45, 140.03, 135.72, 132.47, 131.04, 128.87, 119.90, 113.87, 60.99, 51.07, 37.92, 31.98, 22.75, 19.78, 14.18 ppm; ^{19}F NMR (282 MHz, $CDCl_3$, 30 °C): δ = -116.31 (vbs, 3F), -127.44 (vbs, 1F), -129.24 (bs, 1F), -130.18 (s, 2F), -151.61 (s, 1F), -153.70 (s, 1F), -157.29 (s, 2F), -158.36 ppm (s, 3F); UV/Vis (methanol): λ_{max} = 396, 416, 478, 613 nm.

EA: calcld for $C_{40}H_{13}F_{14}N_4O_2SMn$: C, 51.41; H, 1.40; F, 28.46; N, 6.00; O, 3.42; S, 3.43; Mn, 5.88, found: C, 51.29; H, 1.57; F, 28.21; N, 5.88; O, 3.22; S, 3.23. MS (MALDI+) m/z: calcd. for $C_{40}H_{13}F_{14}N_4O_2SMn$: 933.9916 $[M]^+$, 933.9994 $[M+H]^+$, found: 933.9910 $[M]^+$, 934.9986 $[M+H]^+$.

5,10,15-tris(2,3,5,6-tetrafluoro-4-(octadecyloxy) phenyl) corrole

20 mg H_3TpFPC and 30 equiv. sodium hydride (60 %, in mineral oil) were placed in a 3-neck round-bottom flask under argon. THF and 4 equiv. of 1-octadecanol were added and the reaction mixture was refluxed and stirred for 40 min. The reaction was monitored by TLC and after complete conversion 8 ml water were added to the mixture. The organic solvent was evaporated under reduced pressure. 10 ml of a saturated NH_4Cl solution were added to the aqueous phase and the solution was extracted twice with DCM. The combined organic phases were extracted three times with water. The solvent was evaporated under reduced pressure. Purification was performed by column chromatography (silica gel, DCM:heptanes = 2:3) to obtain 37 mg of 5,10,15-tris(2,3,5,6-tetrafluoro-4-(octadecyloxy) phenyl) corrole (95% yield). The metalation procedure was performed as described above.

Analytical data of *5,10,15-tris(2,3,5,6-tetrafluoro-4-(octadecyloxy) phenyl) corrole*: yield = 95 %, ^{1}H-NMR (700 MHz, $CDCl_3$, 25 °C): δ = 9.20 – 8.50 (m, 8H, *β*-H), 4.55 (t, 6H, -O-CH_2 (position 1)), 2.05 (m, 6H, CH_2 (position 2)), 1.87 (m, 6H, CH_2 (position 3)), 1.75 (m, 6H, CH_2 (position 4)), 1.70-1.10 (m, 60H, CH_2), 0.88 (t, 27H, CH_3); ^{13}C-NMR (175 MHz, $CDCl_3$, 25 °C): δ = 147.5, 146.9, 146.1, 145.5, 142.1, 142.3, 141.9, 141.8, 140.7, 140.6, 140.5, 140.4, 138.6, 138.4, 116.8, 113.9, 111.7, 95.1 (147.5–95.1 carbon atoms of the macrocycle), 37.1, 32.8, 32.0, 30.4, 30.2, 30.1, 30.0, 29.8, 29.7, 29.7, 29.5, 29.4, 29.3, 27.1, 25.7, 19.8, 14.4, 14.2, 14.1 (37.1– 4.1 carbon atoms of the aliphatic side chain); ^{19}F-NMR (282.32 MHz, CDCl3, 25°C): δ = -139.5 (dd, ^3J = 22.9 Hz, ^4J = 7.9 Hz, ^{2}F, F*o*), -140.0 (dd, 3*J* = 22.0 Hz, 4*J* = 7.3 Hz, 4F, F*o*), -157.06 (dd, 3*J* = 22.2 Hz, 4*J* = 7.4 Hz, 4F, F*m*), -157.5 (dd, ^3J = 23.1 Hz, ^4J = 8.0 Hz, ^{2}F, F*m*); HR-MS (APPI) m/z: calcd. for $C_{91}H_{122}F_{12}N_4O_3$: 1547.9395 $[M+H]^+$ found 1547.9395 $[M+H]^+$.

Analytical data of *Manganese-5,10,15-tris(2,3,5,6-tetrafluoro-4-(octadecyloxy)-phenyl) corrole* **2:** yield = 84%, ^{19}F-NMR (282.4 MHz, $CDCl_3$, 25 °C): δ = -116 - -132 ppm (6F), -151 - 155 ppm (6F). UV/Vis (CH_2Cl_2): λ_{max} (ε): 398 (1.8 x 10^4) (shoulder), 419 (2.0 x 10^4), 476 (1.65 x 10^4), 536 (0.63 x 10^4), 575 (0.87 x 10^4), 608 (0.93 x 10^4), 629 (1.1 x 10^4) nm. EA: calcld for $C_{91}H_{119}F_{12}MnN_4O_4$ (1599.8969): C, 68.32; H, 7.50; F, 14.25; N, 3.43; O,

3.00 Found: C 68.07, H 7.38, F, 14.05; N 3.23; O, 2.81. MS (APPI-TOF) m/z: calcd. for $C_{91}H_{119}F_{12}MnN_4O_4$ 1599.8549 $[M+H]^+$, found: $[M+H]^+$.1599.8574.

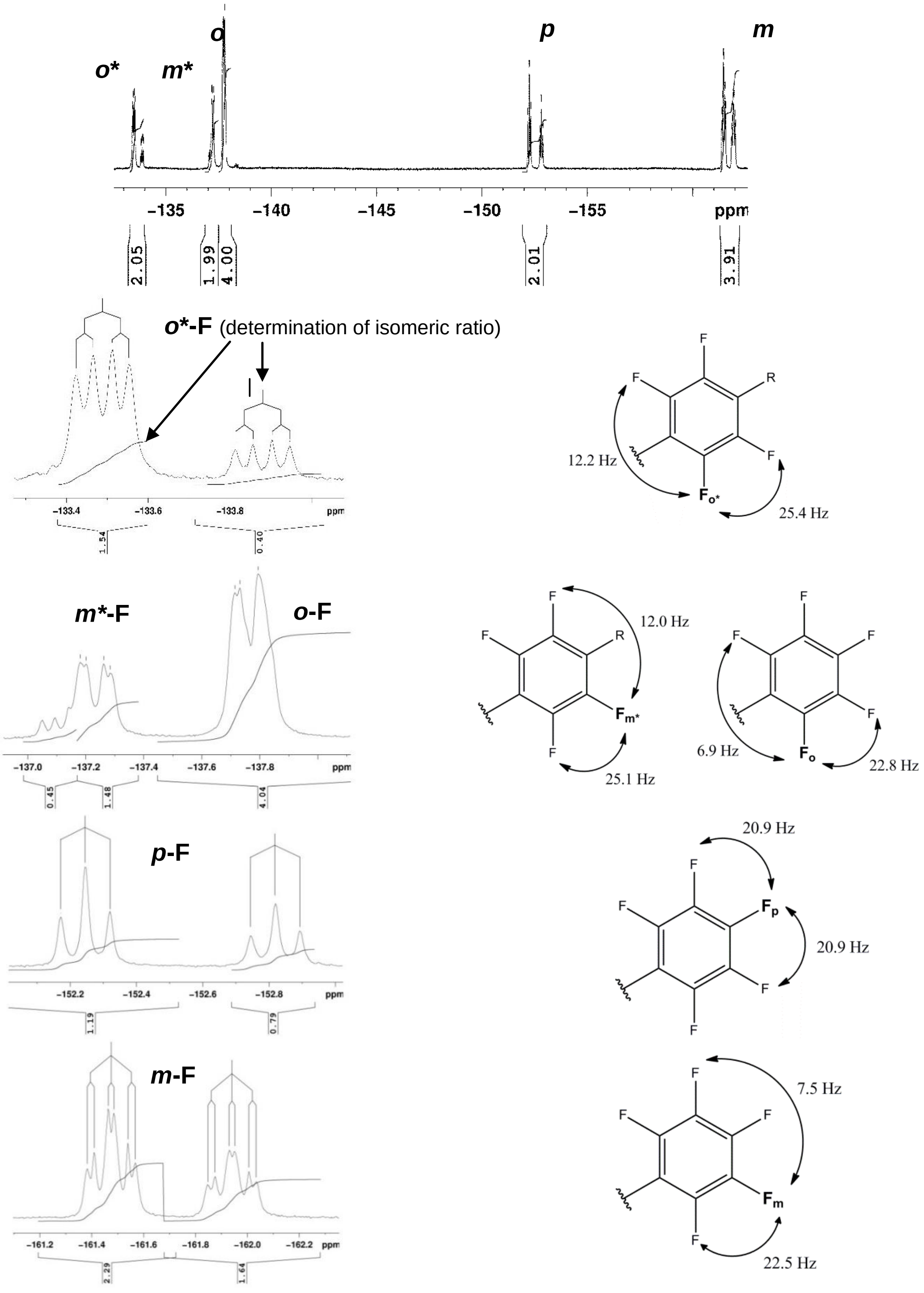

Figure S1: ^{19}F-NMR spectrum of *10,15-bis(pentafluorophenyl)-5-(2,3,5,6-tetrafluoro-4-((carboxyethyl) thio)-phenyl) corrole and 5,15-bis(pentafluorophenyl)-10-(2,3,5,6-tetrafluoro-4-((carboxyethyl)-thio)-phenyl) corrole* the ^{19}F-^{19}F scalar coupling patterns and the determinaton of the isomeric ratio. The asterisks denote the positions on the functionalized moiety.

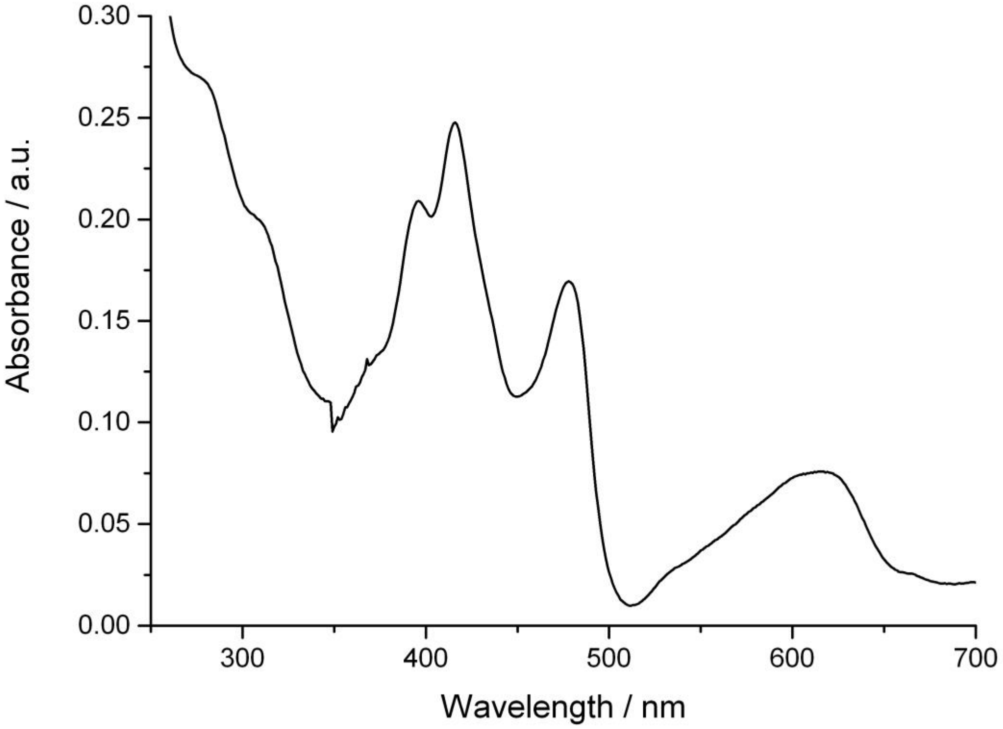
Absorbance / a.u.
0.30
0.25
0.20
0.15
0.10
0.05
0.00
300
400
500
600
700
Wavelength / nm

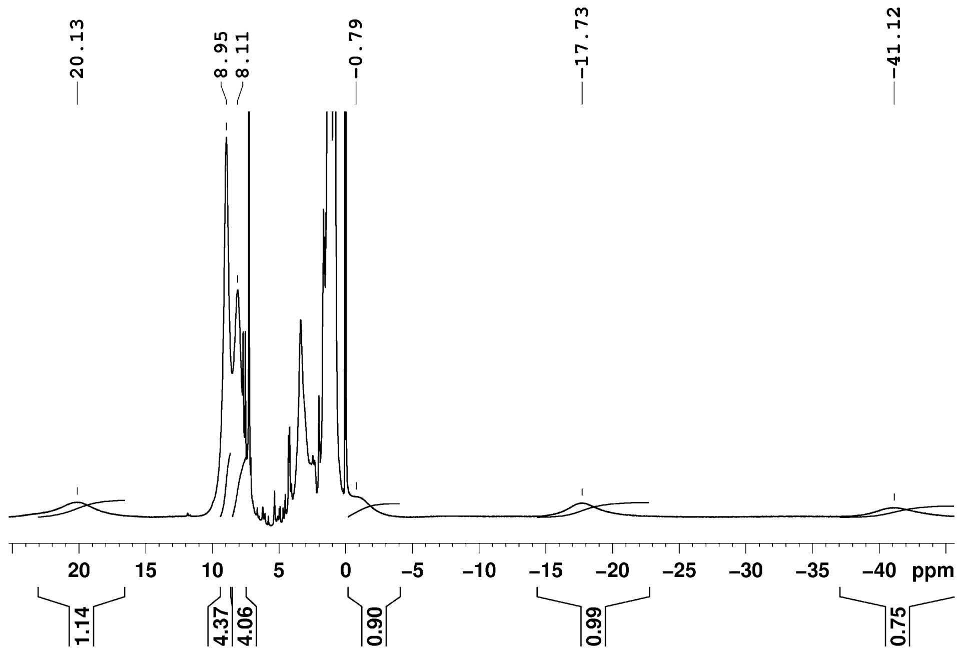
20.13
8.95
8.11
−0.79
−17.73
−41.12
20
15
10
5
0
−5
−10
−15
−20
−25
−30
−35
−40
ppm
1.14
4.37
4.06
0.90
0.99
0.75

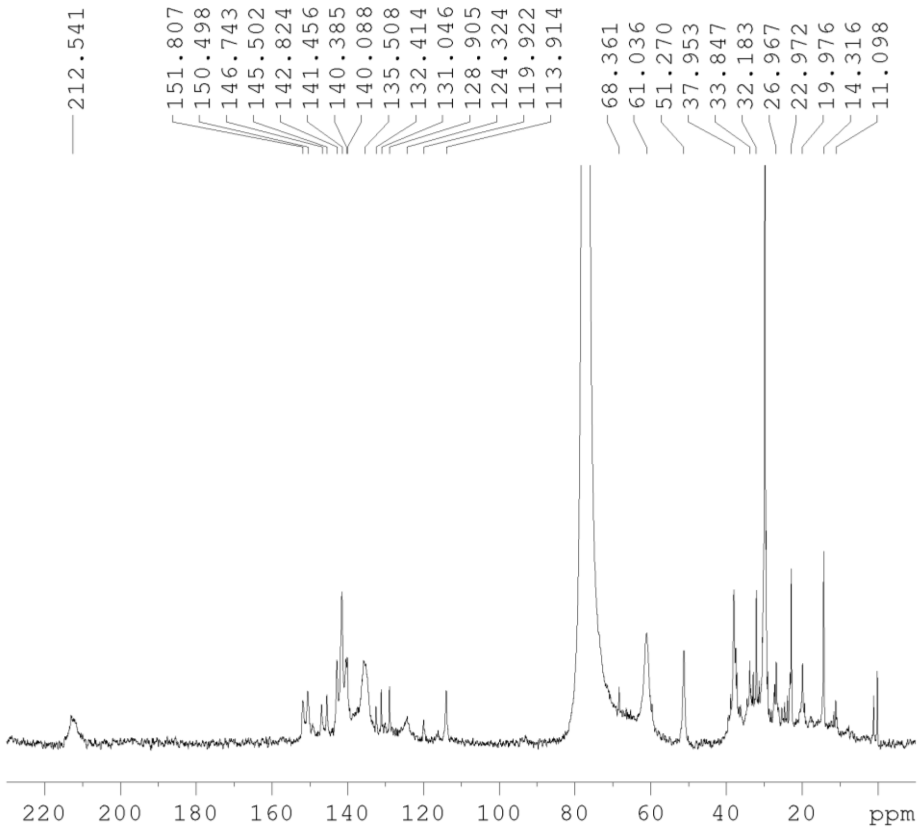

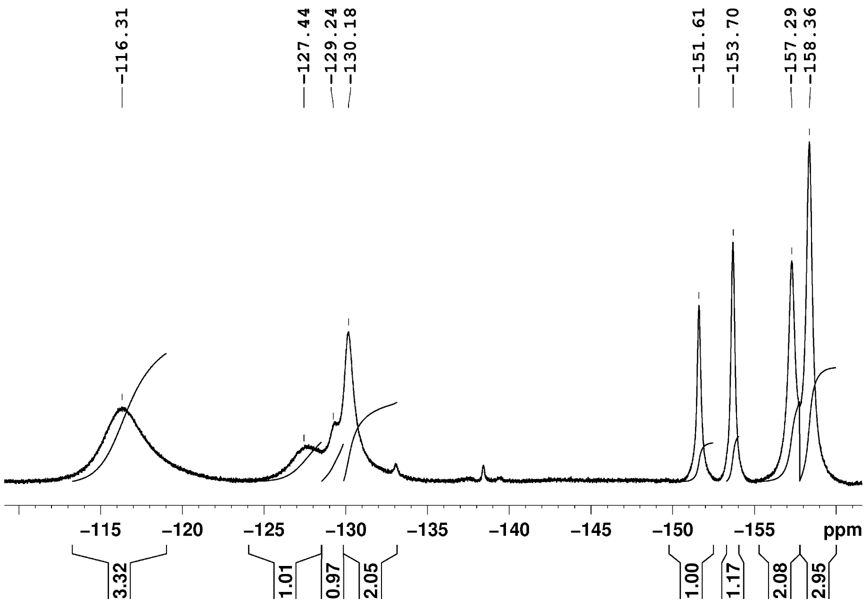

Figure S2: UV-Vis, ^{1}H, ^{13}C NMR and ^{19}F NMR spectra of **1** in $CDCl_3$.

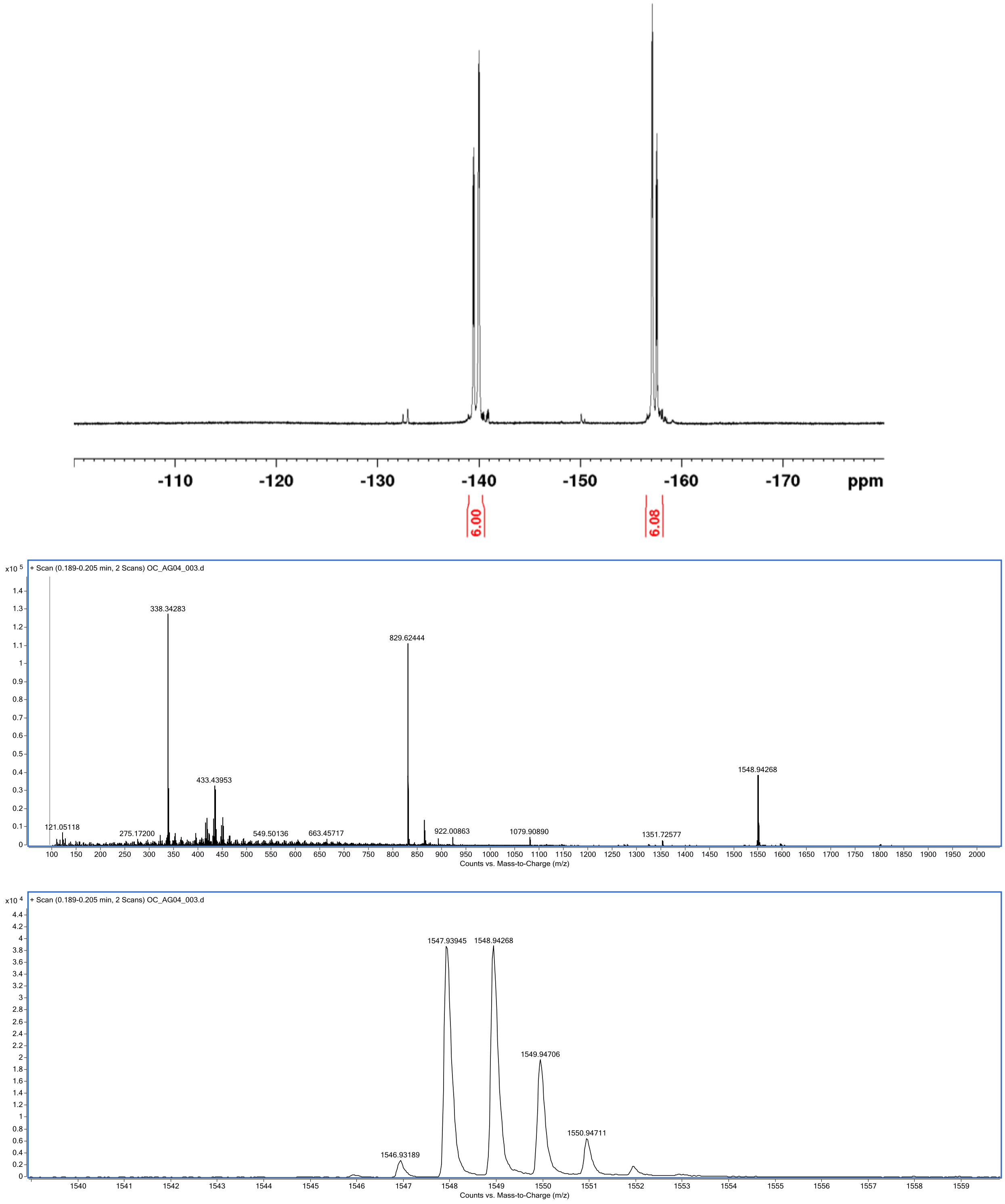

Figure S3: ^{19}F NMR spectrum and high resolution APPI-MS spectra of *5,10,15-tris(2,3,5,6-tetrafluoro-4-(octadecyloxy) phenyl) corrole.*

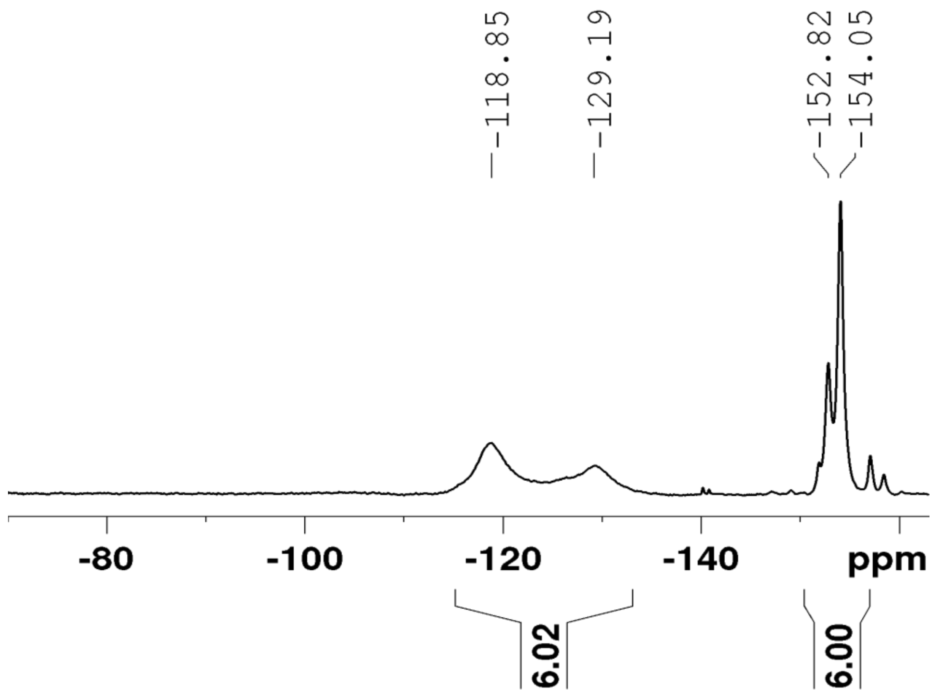

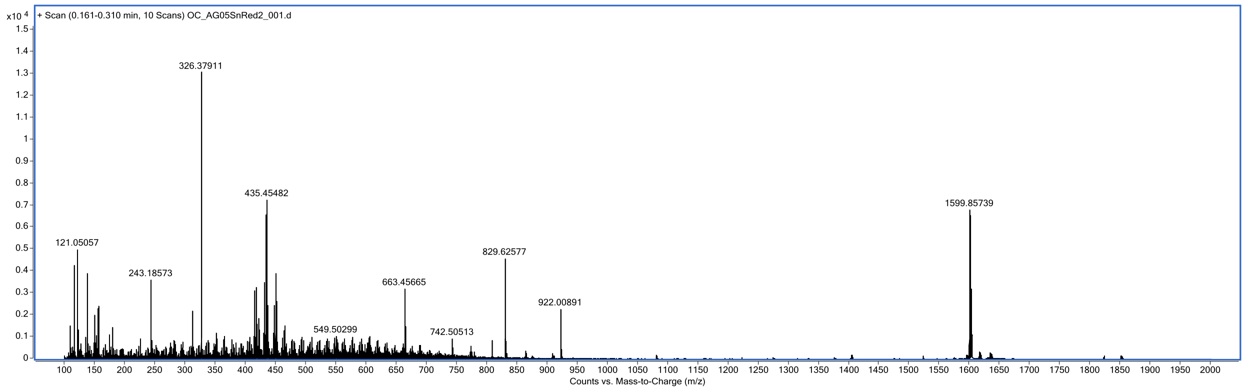

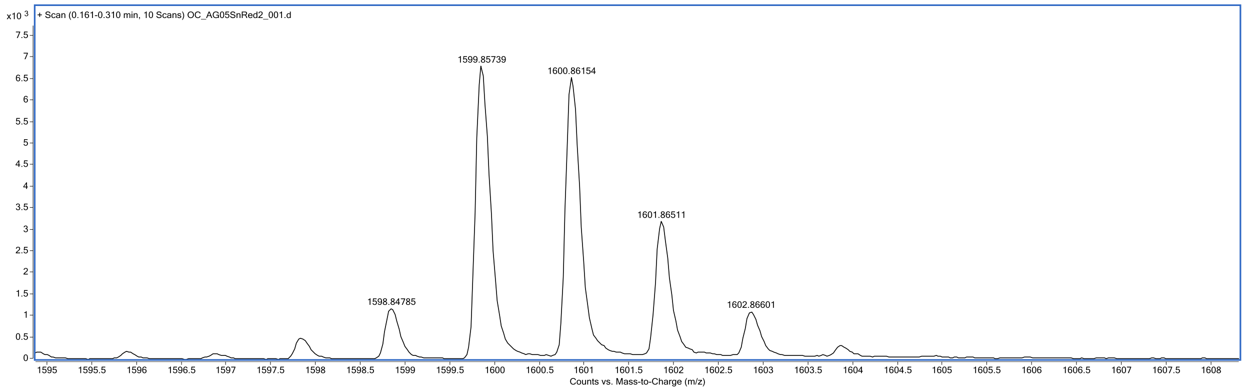

Figure S4: ^{19}F NMR spectrum and high resolution APPI-MS spectra of corrole **2**.

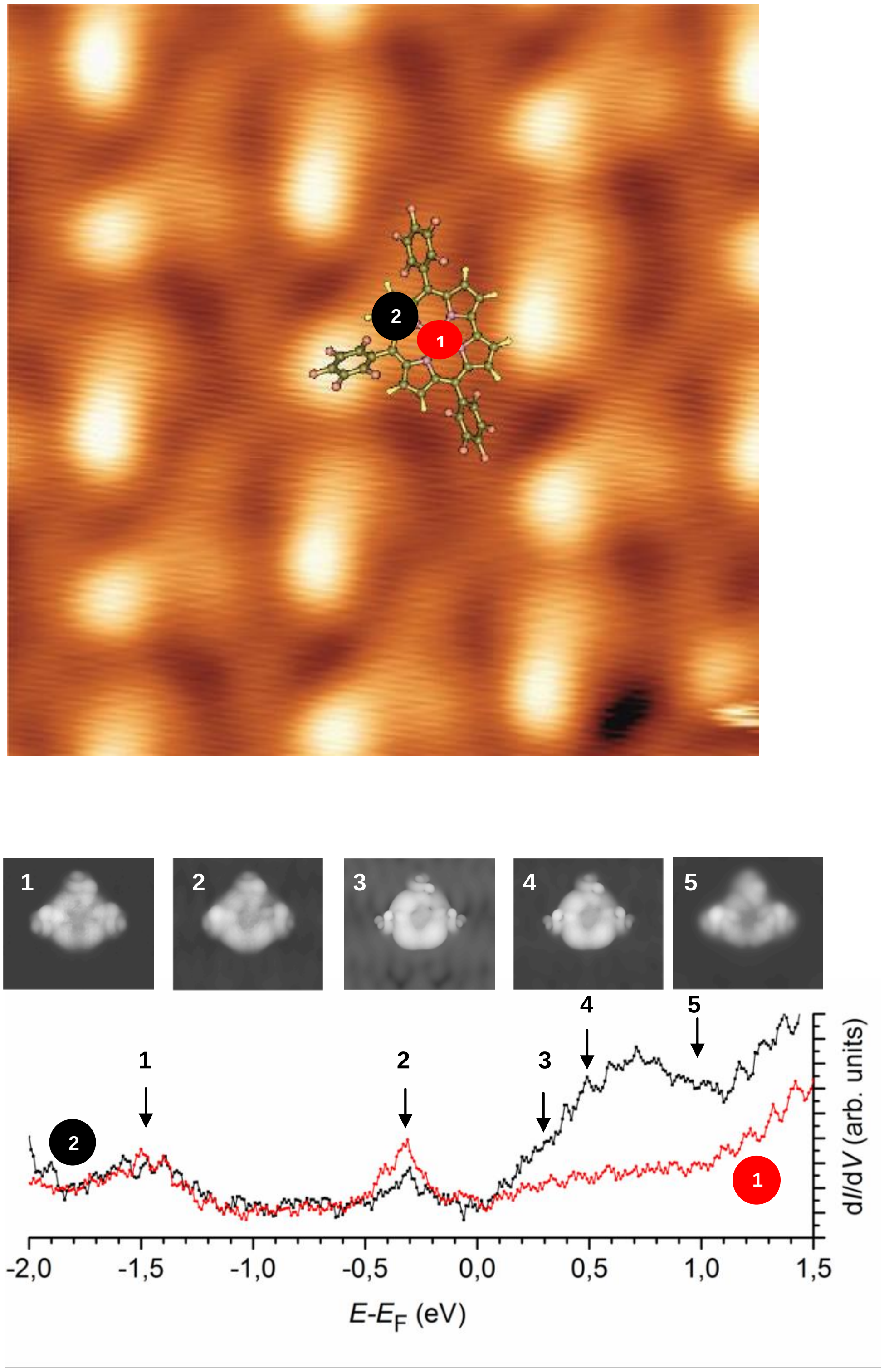

Figure S5: STM image (top), dI/dV spectra (bottom) (1) STM tip at the center and (2) STM tip at the macrocyclic region of a single manganese corrole **3** (adsorbed on Ag(111) surface at 7 K) and simulated STM images at bias voltages of -1.5 V, -0.3 V and 0.5 V, using the Tersoff-Hamann model.

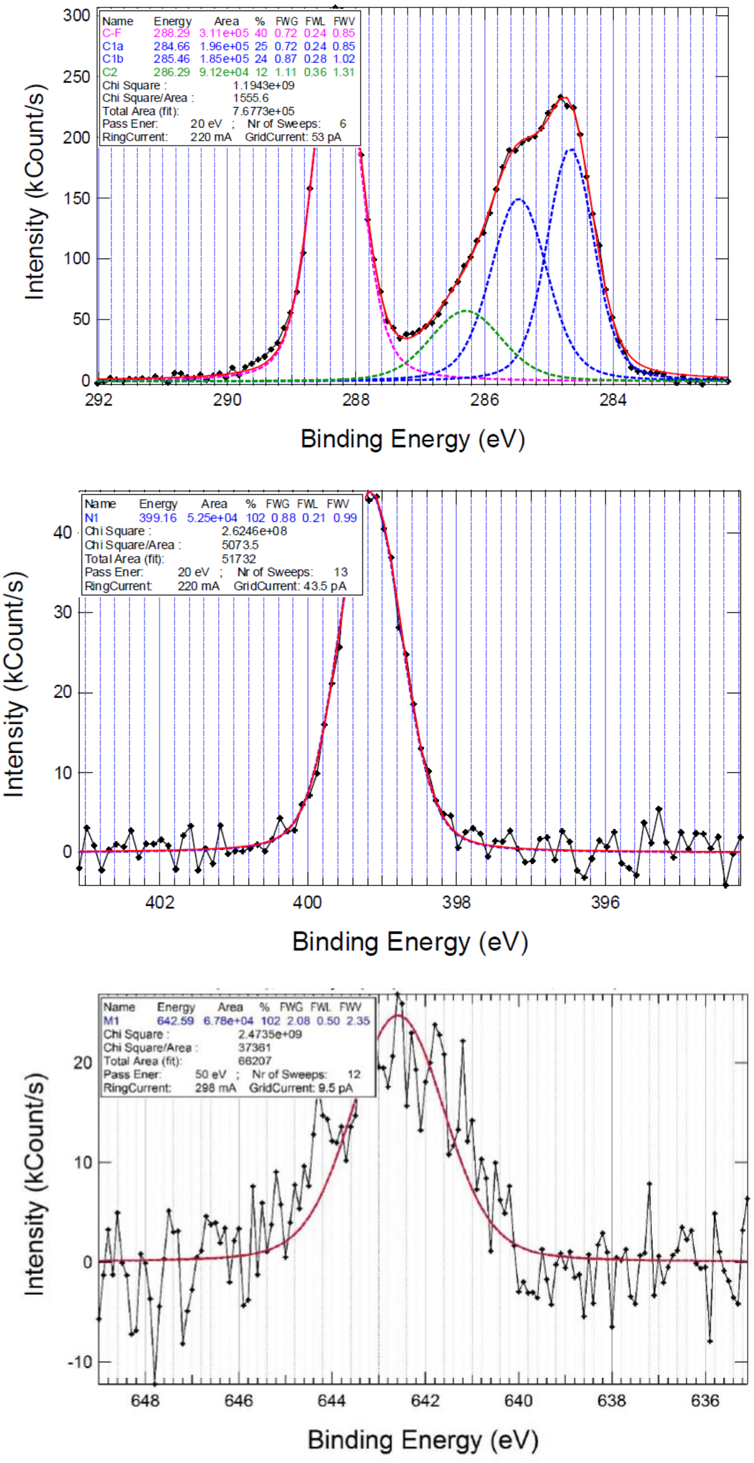

Figure S6: XPS spectra of a) C 1s b) N 1s and c) Mn 2p for the monolayer of Mn(TpFPC) **3** on Ag(111).

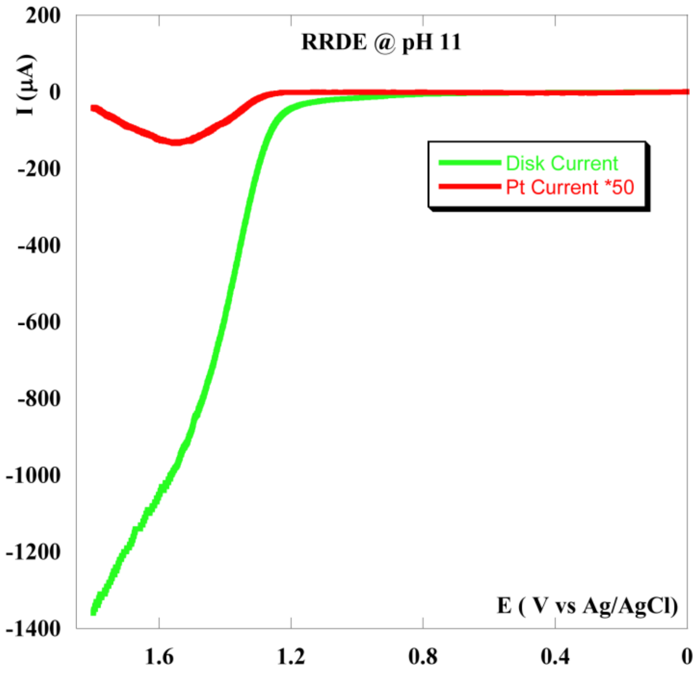

Figure S7: RRDE data of partially oxidized species during oxygen evolution reaction.

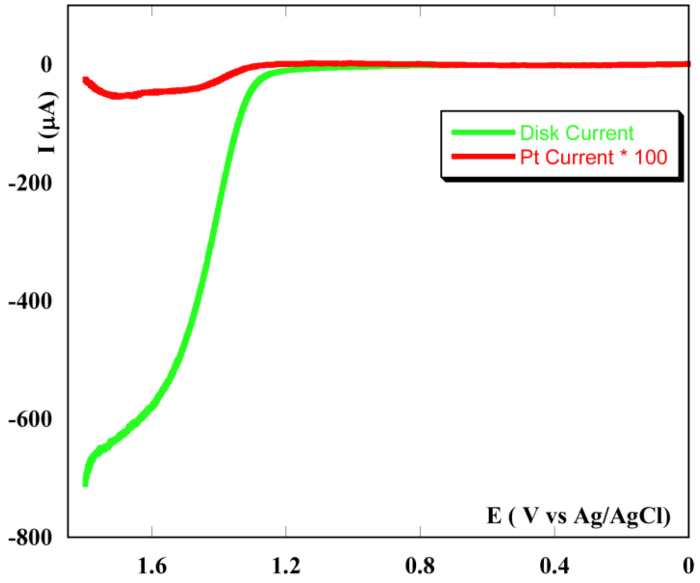

Figure S8: RRDE data of the catalyst **1** immobilized on EPG electrode in pH 11.0 buffer at a constant rotation of 300 rpm and 10mVps scan rate holding platinum at a constant potential of 0.3 V where platinum oxidized the produced partially oxidized species in the process of oxygen evolution.

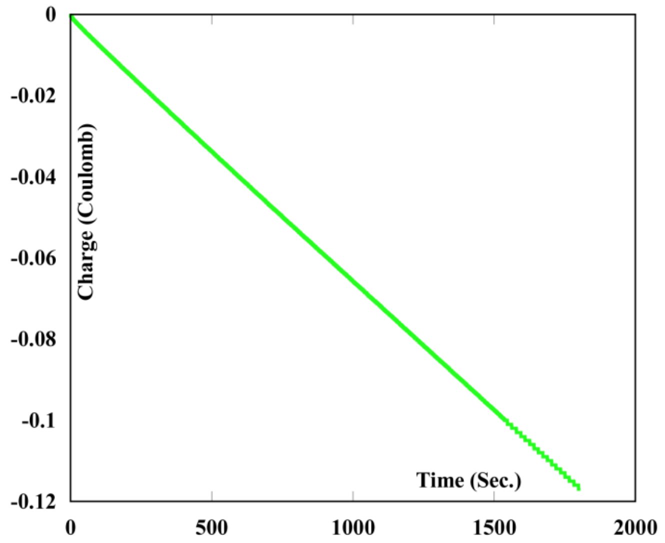

Figure S10: Determination of the turnover number (TON) by heterogeneous bulk electrolysis at a controlled potential of 1.4 V vs. Ag/AgCl for 11.1 hours.

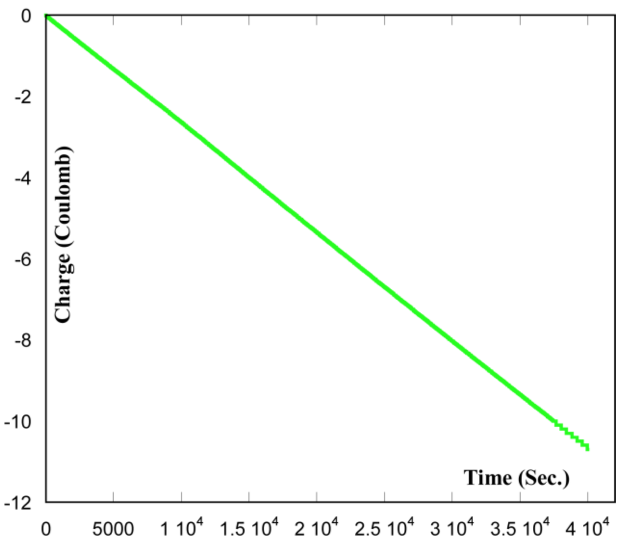

Figure S10: Determination of Faradaic efficiency by the inverted burette technique.

References:

[1] Reith, L. M.; Stiftinger, M.; Monkowius, U.; Knör, G.; Schoefberger, W., *Inorg. Chem.* 2011, *50*, 6788–6797.
